# Evolution of Prospective Secondary Education Economics Teachers’ Personal and Emotional Metaphors

**DOI:** 10.3389/fpsyg.2021.606153

**Published:** 2021-03-26

**Authors:** Lucía Mellado, Laura Parte, Susana Sánchez-Herrera, María Luisa Bermejo

**Affiliations:** ^1^Department of Business and Accounting, Faculty of Economics and Business Administration, National Distance Education University (UNED), Madrid, Spain; ^2^Department of Psycology and Anthropology, Faculty of Education, University of Extremadura, Badajoz, Spain

**Keywords:** evolution of personal and emotional metaphors, economics, prospective secondary education teacher, beliefs, educational models, emotions

## Abstract

This study examines personal and emotional metaphors of prospective economics teachers about the roles they themselves as teachers and their pupils would play by analysing their drawings and responses to open questions. This is a longitudinal study that analyses the evolution of future instructors using two periods: before and after their teaching practicum. Metaphors are categorised into four classes: behaviourist/transmissive, cognitivist/constructivist, situative/socio-historical, and self-referential. The categories for emotions are primary or social and positive, negative, or neutral. The results show that the highest percentage of metaphors for the teacher’s role in both questionnaires were cognitivist/constructivist. Comparison of the findings before and after the teaching practicum revealed no changes in most of the participants’ metaphors and associated models. The analysis also reveals that among those who change, the tendency is to evolve towards more pupil-centred metaphors and associated models. The most common pupil metaphors are behaviourist and cognitivist, increasing after the practicum. Finally, most of the emotions expressed are positive and social, also increasing after the practicum.

## Introduction

In 1985, the Joint Council on Economic Education, 1985 of the United States expressed its concern for the need to help teachers teach Economics and highlighted teacher training as a key element in this process. Salemi et al. (2001) insist on initial teacher training as being a step prior to implementing methods of teaching Economics that are based on active learning on the part of the pupils. In the interest of improving the training of Economics teachers, Salemi (2003) proposes a training model that offers ideas about the objectives, content, and methods of teaching the subject and emphasizes the contribution of practice teaching for prospective teachers. Since the 1990s, different lines of research have been developed in economics education, one of them being the study of the concepts and practice of Secondary Education Economics teachers (Travé and Pozuelos, 2008). Understanding the factors that favour or hinder the training and professional development of teachers is an essential element in planning and putting into practice training programs that result in improved teaching and learning in class (Estepa and Cuenca, 2007).

For Molina and Travé (2014), the incorporation of the subject of Economics into Compulsory Secondary Education requires configuring a specific Economics Education, within the general area of Social Sciences Education, to base the initial and ongoing training of teachers in this field on it. However, when prospective secondary teachers begin their Master’s course, they do so not only with the knowledge they have acquired in their speciality, but also with ideas, attitudes, and feelings about education, which are usually deeply rooted because of the fruit of the many years they themselves had spent at school, either accepting or rejecting the roles that they saw their teachers as having played (Mahlios et al., 2010). For Löfström and Poom-Valickis (2013), prospective teachers’ beliefs about the teacher’s role are based on their own school experiences as pupils. They do not easily change their beliefs, and even less so their educational practices because overall educational change is a complex process involving numerous obstacles and impedances (Vázquez et al., 2012). Metaphors provide an indirect way to approach teachers’ beliefs about teacher’s role, which may be implicit or hidden (Löfström and Poom-Valickis, 2013). Metaphor is a powerful cognitive tool in gaining insight into teachers’ beliefs (Wan et al., 2011). This reflection about metaphors may help change their teaching roles, usually constructed throughout their schooling. The analyses of the emotions reflected in the metaphors is also a matter of concern. As Day (1999) suggests, change is not just a matter of the head, but also of the heart. It will be difficult to put changes into effect unless they are compensated affectively.

In this article, we present a descriptive study of a sample of prospective Secondary Education Economics teachers, which analysed the evolution of their personal metaphors about themselves as future teachers and about their pupils’ role from before their teaching practicum to after it, as well as the emotions associated with those metaphors. This study was a result of a bigger inter-disciplinary research project conducted by an inter-university team, which investigates teachers of different specialties and levels. Although the samples and the methodology are specific for each study, the theoretical framework is common and has already been described in previous works (Mellado et al., 2016, 2018).

### Pre-service Teachers and Metaphors

A metaphor is a symbolic way of describing an idea or concept by replacing it with another word or sentence that has a certain objective or subjectivity similarity for the user of the metaphor. Metaphors are an indispensable mechanism of the mind by which people piece together and create new meaning. They help structure a highly relevant dimension of individual’s perceptions and conceptual system (Lakoff and Johnson, 1980).

Teachers often use symbolic and metaphoric language when speaking about their professional ideas and practices (Lakoff and Johnson, 1980). Every teacher elaborates a particular body of professional practical thinking as a result of their own individual activity and relations with their community. It is difficult to access this thinking and provide it with sense since educators have certain views about their vocation, which are tough to communicate in an organised manner.

Metaphors articulate teachers’ thoughts and create connections between practical knowledge and in-class life narratives (Buaraphan, 2011; Zhao et al., 2011; Hamilton, 2016). They help give an overall organization and articulation to a teacher’s conceptions, roles, and practical knowledge and uncover the implicit referents, which sustain that teacher metaphors have a powerful influence on their teaching behaviour in class (Tobin et al., 1994; Boujaoude, 2000).

Numerous studies have used teachers’ metaphors as a methodological tool to analyse their beliefs about teaching and learning (Guerrero and Villamil, 2002; Cassel and Vincent, 2011; Szukala, 2011; Duru, 2015; Seung et al., 2015; Kavanoz, 2016). Previous researches with a focus on pre-service secondary teachers of different specialties (Stofflett, 1996; Mellado et al., 2012; Mellado et al., 2016, 2018) show that the metaphors were not associated with specific content, but were an expression of a general vision of teaching and the role of the teacher, formed from the students’ own experiences as pupils at school (Briscoe, 1991).

Therefore, metaphors are a significant matter of study in education research and are potent instruments that help inspire pre-service teachers’ reflection (Paavola and Hakkarainen, 2005). Furthermore, they make possible a holistic understanding of what happens in the classroom (Buaraphan, 2011), facilitating to promote links with past experiences (Zhao et al., 2011; Hamilton, 2016).

Lakoff and Johnson (1980) noted that metaphors are essential for education because they can model social realities and become a guide for prospect actions that will adjust to those metaphors: ‘*In this sense metaphors can be self-fulfilling prophecies’* (p. 156). Through a process of critical reflection, teachers are able to build new roles and change their pedagogical conceptions and teaching practices, while embracing new metaphors, which are compatible with those changes and consistent with the educational styles they want to adopt (Sillmam and Dana, 2001; Heston and Veenstra, 2002; Russell and Hrycenko, 2006; Pinnegar et al., 2011; Thomas and Beauchamp, 2011).

While the literature on teachers’ metaphors is extensive, there is still room for progress at least because the analysis of teachers’ use of personal metaphors is an effective and extraordinary tool to understand educators’ thinking and behaviour in class (Shaw et al., 2008). For instance, Mahlios et al. (2010) state that there have been few longitudinal studies on how instructors’ metaphors evolve over time.

Saban (2006) reviewed previous literature on teachers’ metaphors and suggested that educators might use metaphors to help their students reflect on their beliefs and values about learning and teaching and on how metaphors change during their professional practice. Seung et al. (2015) also call the use of metaphors as an intervention tool. Therefore, the analysis of their own personal metaphors by pre-service teachers before and after their practicums is important because these metaphors may motivate reflection and can help the students comprehend and self-regulate their roles and be conscious of the intricacies involved in changing those images (Leavy et al., 2007; Seung et al., 2011; Tannehill and MacPhail, 2014; Gosselin and Meixner, 2015).

### Emotions and Metaphors

There are emotional aspects that are irrational from a cognitive perspective, but which nevertheless impact teachers’ decision making, actions, and conceptions (Buaraphan, 2011). Emotions are mostly essential for pre-service teachers (Hargreaves, 2005) since, at the beginning of their professional lives, they are establishing their teaching approaches, class routines, and strategies. This is particularly important given that, as has been observed in other studies (Sillmam and Dana, 2001), their own time as pupils in school has a strong influence on their conceptions and models of teaching. According to Ng et al. (2010), the first teaching experience changes their beliefs to be more focussed on self rather than students, but other emotional beliefs remain (good teachers being kind, caring, understanding, and charismatic, who assist their students to achieve their goals). Brígido et al. (2013) show that the emotions pre-service teachers felt towards teaching certain subjects during the practicum are related to those they felt when they learned those contents in secondary school. Oosterheert and Vermunt (2001) included the regulation of emotions as a functional component of learning to teach. Teacher training is a stage during which these aspects need to be considered so that prospective teachers will be able to control and self-regulate their emotions. Teaching in the practicum is mainly experienced as emotionally positive by the student to be prospective teachers (for example, emotions like interested, enthusiastic, attentive, satisfied, awaked, elated, etc.), although certain negative emotions (for instance, anxiety, nervousness, worry) are also prevalent during the practicum (Hascher and Hagenauer, 2016).

In this article, we based the definition of emotions proposed by Bisquerra (2000, 63):

Emotions are reactions to the information we receive in our relationships in the environment. The intensity of the reaction depends on subjective assessments that we make of how this information will affect our well-being. These subjective assessments will involve prior knowledge, beliefs, personal objectives, perception of a challenging environment, etc. An emotion depends on what is important to us.

However, emotions are also produced by the recall or evocation of events that occurred in the past (Damasio, 2010) or the anticipation or expectation of possible future situations (Aguado et al., 2011).

Metaphors are a bridge between affect and cognition. Consequently, they help teachers be aware of their feelings. As noted by Zembylas (2004), teachers’ metaphors are especially well suited for expressing their emotions. These metaphors have a major affective component since teachers construct them based on their personal experience. For Eren and Tekinarslan (2013), the affective aspects of the prospective teachers’ metaphors should be considered during teacher education to understand how they feel about crucial aspects of the teaching and learning-related processes. In addition, Wegner et al. (2020) considers emotions as an important variable related to metaphor about learning.

### Objectives

This article analyses the evolution of prospective Secondary Education Economics teachers’ personal and emotional metaphors on teaching and learning. Pre and post practicum questionnaires were used to elicit metaphors and drawing metaphors. The specific objectives are as follows:

1. To describe the nature of personal and emotional metaphors used by teachers, divided into four main categories: behaviourist/transmissive, cognitivist/constructivist, situative/socio-historical, and self-referential.
2. To study the evolution of their personal and emotional metaphors about the teacher’s and the pupils’ roles before and after their practicum.
3. To determine the evolution of their teaching models associated with metaphors.
4. To analyse the emotions reflected in the emotional metaphors.

## Materials and Methods

### Sample

Spanish Secondary Education teachers are required to hold a previous four-year university degree, which is not oriented towards teaching and a Master’s degree course in Secondary Teacher Education – a one-year postgraduate course aimed at the educational preparation of the prospective Secondary Economics teacher. In the first semester, this postgraduate Master’s degree includes courses on the psychology of learning, pedagogy, and teaching methods specific to the corresponding specialty, as well as a three-month practicum in a secondary school at the end of the first semester.

The study participants were 27 pre-service teachers of a Secondary Education Teaching Master’s course who were specializing in Economics. The academic years were the 2012/2013 and 2013/2014 with samples of 14 (from E1 to E14) and 13 (from E15 to E27) students, respectively. In previous works (Mellado et al., 2017a), we have analysed different aspects of the 2012/2013 course sample (from E1 to E14). In this work, we extend the sample to two academic years.

Table 1 shows the characteristic of the sample participants. The number of participants is 27 (*N* = 27). By gender, there were 9 men (33.3%) and 18 women (66.6%), and by age, 51.9% were aged between 21 and 25 years, 29.6% were 26 to 30 years, and 18.5% were 31 years or older. By type of degree with which the participants entered the Master’s course, most had a (Bachelor’s) Degree in Business Administration (DBA) (51.9%), followed by Economics (25.9%), Tourism (18.5%), and Law (7.4%). Finally, 4 participants (14.8%) had teaching experience and 23 (81.2%) did not.

**TABLE 1:** Characteristics of the sample participants.

	Gender	Age	Teaching		Gender	Age	Teaching
E1	M	21–25	No	E15	M	21–25	No
E2	W	21–25	No	E16	M	26–30	No
E3	W	26–30	No	E17	M	21–25	No
E4	W	26–30	No	E18	M	21–25	No
E5	W	>35	Yes	E19	M	21–25	No
E6	W	21–25	No	E20	M	>35	No
E7	W	21–25	No	E21	W	31–35	Yes
E8	W	21–25	No	E22	M	21–25	No
E9	W	21–25	No	E23	W	26–30	No
E10	W	21–25	No	E24	W	>35	No
E11	W	21–25	No	E25	W	26–30	No
E12	W	21–25	No	E26	W	26–30	No
E13	W	31–35	Yes	E27	W	26–30	Yes
E14	M	26–30	No				

During the courses, participants were informed about the study’s objectives and participation was voluntary. To monitor their progress, a questionnaire was conducted before and after the accomplishment of their practicums in secondary schools. In our study, we complied with the ethical procedures for research with human participants. All study subjects were guaranteed confidentiality and anonymity.

### Data Collection and Qualitative Analysis

In the methodology, we followed Low’s (2015) approach that considers seven steps for a methodological validation: (1) preparing participants, (2) to elicit metaphors, (3) to classify metaphors into educational categories, (4) to connect with educational theories or orientation, (5) to establish behaviour stemming from educational orientation, (6) to define a plan for future action, and (7) to evaluate the change in metaphors.

The research team had previous experience through prior work on the metaphors of secondary school teachers of different specialties (Mellado et al., 2012). The data were collected using an anonymous questionnaire. Participants were asked some questions about personal information (gender, age, and previous undergraduate degree) and open questions about their own personal metaphors as teachers and about the metaphors that they identified with pupils’ learning and the reasons which led them to choose these metaphors. The use of written prompts or metaphor statements is an efficient way to analyse teacher conceptions about teaching and learning; however, these methods have been criticized for their heavy reliance on the statements to explain the implicit teacher conceptions (Seung et al., 2015). The questionnaire was based on Leavy et al. (2007) and Martínez et al. (2001) as well as our previous studies (Mellado et al., 2016, 2017b). The items about metaphors in the questionnaire are as follows:

1. When you teach in a secondary school classroom, what metaphors would you identify yourself with?
2. Explain the reasons that led you to identify yourself with those metaphors.
3. With what metaphors do you identify pupils in relation to learning?
4. Explain the reasons why you identify pupil learning with these metaphors.
5. Try to make a drawing to better symbolise your metaphors as a teacher and your pupils’ relationship with the learning process.

We included metaphors’ drawings in the data collection procedures, an instrument validated by prior literature with prospective teachers (McGrath, 2006; Buaraphan, 2011; Löfström et al., 2015). Markic and Eilks (2015) point out that pictures made by educators are like mirrors of their professional identity and may help identify whether a class is teacher-and-content centred or pupils-and-learning centred. Weber and Mitchell (1996) consider that an image has a great potential to express metaphors. Sumsion (2002) used teachers’ own drawing to analyse the changes in the professional identity in a beginning teacher. Before giving the participants the survey, we informed them about the significance of metaphors in the educational context. Nevertheless, we did not present any examples of personal metaphors so as not to condition their answers.

To categorise the personal metaphors of the prospective teachers, we leaned on Leavy et al.’s (2007) four classes of metaphors: behaviourist/transmissive – with a passive pupil whose motivation is extrinsic, the instructor as a source of content, and with the distinctive of the classes being centred on the instructor and information; cognitivist/constructivist – with active learners that build their knowledge, an individual process in which the stimulus does not depend on external reinforcement, and with the educator as a helper; situative/socio- historical – where the focus is on the social context in which learning is constructed and learners’ motivation arises from the participation in the activities with the educational community; and self-referential – comprising particular metaphors centred on what teaching means for the individuals, with no allusion to components of the exercise of teaching. The meaning that participants gave to the metaphors determined their categorization (Roth, 1993). As Wan and Low (2015) point out, our approach to analyse the elicited metaphors by grouping them into categories has limitations, since the assignment of categories can vary markedly among researchers. In the descriptive analysis, we show the frequencies in each category. To assign a metaphor to one of the categories, the drawings and the reasons given by the participants were examined and discussed among the researchers, contrasting the results with previous studies. Seung et al. (2011) also used comparative methods to analyse teachers’ metaphors.

Our analysis of the metaphors’ evolution and the changes of the students’ didactic models is based on Vázquez et al.’s (2012) progression hypothesis. The evolution from behaviourist/transmissive to constructivist/cognitive and from there to situative/socio-historical was a progression, and the contrary a regression since pupil-centred orientations are an indicator of the implementation of inquiry-based innovative teaching strategies in class, as the National Research Council (1996) advocates. Buchanan (2015) also takes teacher-centred or pupil-centred models as referents for categorization. We take any progression as important in which the prospective teacher changes from behaviourist/transmissive models to cognitive/constructivist or situative/socio-historical ones (both of which express pupil-centred classes). Furthermore, we consider progression to be small when it changes from the cognitive/constructivist category to the situative/socio-historical one. Self-referential metaphors have a very particular meaning that need to be considered specifically in each case. To elaborate Table 2 on the evolution of the models, we analysed all the metaphors expressed by each participant, following the above criterion of progression/regression/no change. When a participant expressed metaphors of different categories, the category of the dominant metaphors was considered, for which the coherence between the text and the drawings is considered.

**TABLE 2:** Evolutions in the teachers’ models before and after the teaching practices (*T*, behaviourist/transmissive; C, cognitivist/constructivist; S, situative/socio- historical; R, self-referential).

Participants	Pre-test to post-test evolution of the teacher metaphors	Changes in the teacher metaphors	Pre-test to post-test evolution of the pupil metaphors	Changes in the pupil metaphors
E1	T – T	No change	T – T	No change
E2	T – T	No change	T – T	No change
E3	T – T	No change	T – T	No change
E4	T – T	No change	R – R	No change
E5	C – C	No change	C – C	No change
E6	C – C	No change	C – C	No change
E7	S – S	No change	R – R	No change
E8	S – S	No change	C – C	No change
E9	S – S	No change	C T – T	Regression
E10	T – S	Progression	R – R	No change
E11	T – CS	Progression	R – R	No change
E12	T – S	Progression	C – C	No change
E13	TC – C	Progression	T – C	Progression
E14	C – S	Progression	C – S	Progression
E15	C – C	No change	T – T	No change
E16	C – S	Progression	C – S	Progression
E17	T – T	No change	T – T	No change
E18	S – C	Regression	T – T	No change
E19	CS – C	No change	R – R	No change
E20	T – T	No change	T – T	No change
E21	SC – SC	No change	SC – C	No change
E22	C – S	Progression	CT – S	Progression
E23	S – C	Regression	ST – CT	No change
E24	R – R	No change	C – C	No change
E25	R – C	Progression	C – C	No change
E26	SC – C	No change	SC – C	No change
E27	SC – S	No change	C – C	No change

After examining the pre-test, and before the practicum, we had a group session with the participants in which, using Power Point, we showed them the metaphors and the drawings that had been made to represent them and discussed their meanings. For Tobin and Fraser (1989), the introduction of a variety of metaphors during initial teacher training allows the prospective teachers to gain a view of the potential of metaphors as a tool, to reflect on their own metaphors, and to develop new metaphors consistent with the teaching models they want to implement (Pinnegar et al., 2011). The post-test was conducted after the teaching practicum. Since there were no longer any regular class sessions in any of the Master’s course subjects, we collected these in one of the tutoring sessions about those practicums.

As Sutton and Wheatly (2003) point out, teachers’ emotions can be analysed through the metaphors they use. In addition, Chen (2003) incorporates emotional metaphors, as teachers describe teaching as feelings, passion, imagination, interest, and creativity.

With respect to discriminating between different types of emotions, there is a lack of consensus among authors. Therefore, we classified the emotions into positive, negative, or neutral and primary or social (Damasio, 2010; Brígido et al., 2013; Hascher and Hagenauer, 2016). Fernández-Abascal et al. (2001) point out that positive emotions involve pleasant feelings, of short duration, and require the mobilization of few resources; negative emotions involve unpleasant feelings, and coping with them requires the mobilization of many resources. Primary emotions are innate and universal (fear, anger, sadness, disgust, and happiness), while secondary ones need a social context, since they are acquired in interaction with others (shame, guilt, pride, enthusiasm, satisfaction, trust, contempt, etc.).

In previous studies (Brígido et al., 2013; Mellado et al., 2016, 2018), we analysed the teacher’s emotions, grouped in primary/social and positive/negative. To assign an emotion to one of the categories, the metaphors’ drawings and the reasons given by the participants were examined and discussed among the researchers.

## Results

### Metaphors’ Evolution and Associated Teaching-Learning Models

The results are not intended to be generalizable because this was not a statistically representative sample. They need to be seen from an interpretative perspective, seeking the meanings of the participants’ individual metaphors.

**CHART 1:**
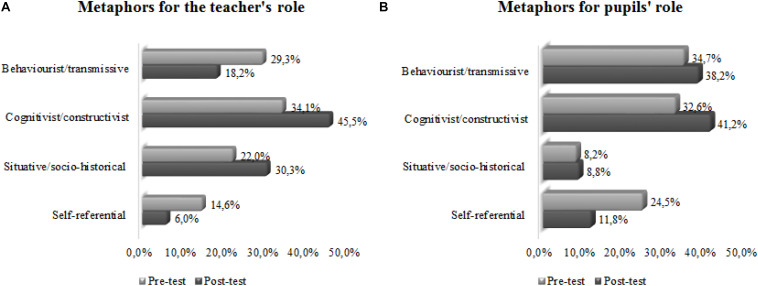
**(A,B)** Comparison of the percentages of metaphors for the teacher’s and pupil’s roles, before and after the practicum.

The 27 participants used 41 metaphors for the teacher’s role in the pre-test and 31 in the post-test. Chart 1A shows the comparison of the percentages of metaphors in the four categories of study, before (pre-test) and after (post-test) the practicum. In both questionnaires, the highest percentage of teacher metaphors were cognitivist/constructivist. Comparison of the results before and after the practicum showed that the teacher metaphors changed from teacher-centred models (the behaviourist/transmissive category) to others more pupil-centred (cognitivist/constructivist and situative/socio-historical categories).

The participants used 49 metaphors for the pupils’ role in the pre-test and 34 in the post-test. Chart 1B presents the comparison of the percentages of metaphors in the four categories of study before and after the teaching practices. Before, the greatest percentage corresponded to behaviourist/transmissive pupil metaphors. However, after the practicum, the greatest percentage corresponded to cognitivist/constructivist metaphors, with both these and the behaviourist/transmissive increasing and the self-referential decreasing. This last finding may reflect a relative lack of definition of the models associated with pupil metaphors, models which begin to be more clearly defined after the practicum. In both questionnaires, the students expressed very few situative/socio-historical pupil metaphors.

By individualizing the models associated with each participant’s teacher and pupil metaphors, we were able to monitor their progress. Table 2 lists the changes observed from pre- to post-practicum for each of the 27 participants. Regarding the teacher’s role, 17 (63.0%) participants showed no change in their metaphors, 8 (29.6%) a progressive change, and 2 (7.4%) a regressive change. Regarding the pupils’ role, 22 (81.5%) of the participants showed no change in their metaphors, 4 (14.8%) a progressive change, and 1 (3.7%) a regressive change. It was notable that 16 participants (59.2%) changed neither their teacher models nor their pupil models.

**FIGURE 1:**
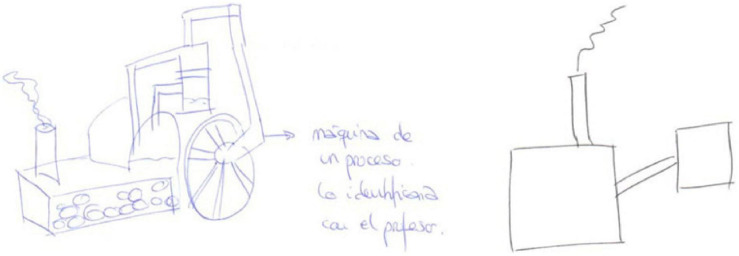
Drawings of E1 for the metaphor of the teacher as machine (pre-and post-test).

Next, we shall highlight some examples. Participant E1 maintained his behaviourist/transmissive metaphors, the teacher as a machine (Alarcón et al., 2014), before and after the teaching practicum (Figure 1). This metaphor is common in studies of business organizations (Morgan, 1980), representing a traditionalist vision of the firm working as a set of machines in a routine, efficient, precise, and predictable way. For the pupils, he maintains a behaviourist/transmissive metaphor of a sponge (Saban, 2010) representing the pupils as absorbing what the teacher gives them.

**FIGURE 2:**
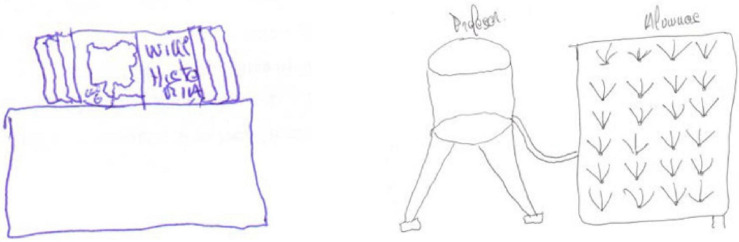
E20’s teacher metaphor drawings as encyclopaedia (in the pre-test) and water tank (post-test).

While Participant E20 changed his metaphors from before to after the practicum, they remained within the behaviourist/transmissive category. Before, the teacher is seen as an atlas or an encyclopaedia (Thomas and Beauchamp, 2011), and after as a water tank that feeds water to the pupils (Figure 2). In both cases the teacher is the transmitter of content and the pupils are passive recipients.

**FIGURE 3:**
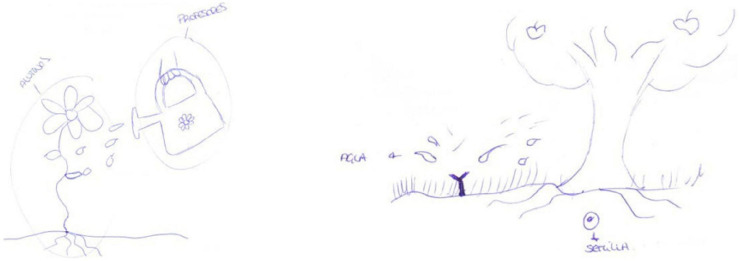
E26’s drawings for the metaphor of the gardener for the teacher and the seed for the pupils (pre-test and post-test).

Participant E26 conserved her metaphors within the cognitive/constructivist category. In both surveys, she identified the teacher with the gardener and the pupils with the seeds that germinate and grow (Figure 3). The gardener must water and care for all the plants according to their needs (Buaraphan, 2011; Kim and Danforth, 2012; Hamilton, 2016), a complement to the metaphor of the pupil as a seed (Gurney, 1995; Saban, 2010).

**FIGURE 4:**
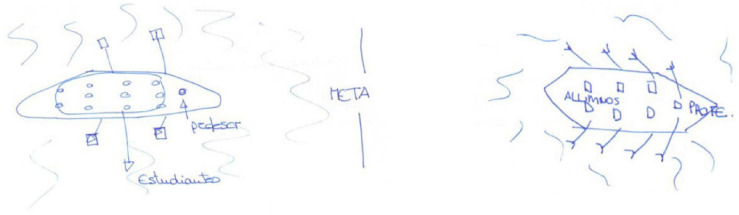
E27’s drawings of a class as rowing crew in the pre-test and in the post-test.

Participant E27 maintained a situative/socio-historical metaphor of a rowing crew both before and after her practicum (Figure 4): ‘all in the same boat rowing in the same direction’. This is a metaphor identified by Buaraphan (2011) in which cooperation is necessary since if there is no cox, then the boat will go off-course; however, if the oarsmen stops rowing, then the boat will stop.

**FIGURE 5:**
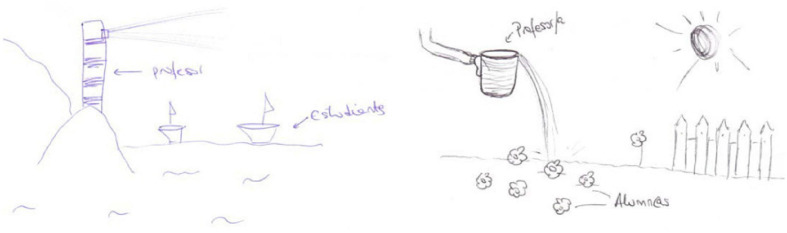
E23’s drawings for the metaphors of a lighthouse that guides boats (pre-test) and watering the flowers (post-test).

Participant E23 expressed a small regression while remaining within the pupil-centred models. In the pre-test, she gave the situative/socio-historical teacher metaphor of a lighthouse (McGrath, 2006), and in the post-test, the cognitive/constructivist metaphor of the teacher watering flowers (Figure 5).

**FIGURE 6:**
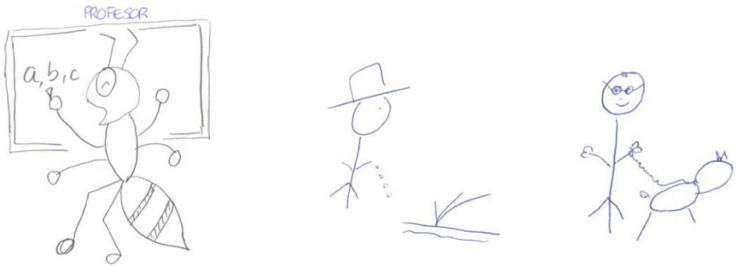
E11’s drawings for the metaphor of the teacher as an ant explaining on the board (pre-test) and as a sower of seeds and a guide dog (post-test).

An example of an important progression in the teacher metaphor is that of E11. In the pre- test, she identifies the teacher with the metaphor of an ant (Saban, 2010), because of its hard-working nature. We classify this as behaviourist/transmissive since the drawing (Figure 6) shows the ant writing on the blackboard with its back to the pupils and without their participation. However, in the post- test her teacher metaphors are those of a sower of seeds (of cognitive/constructivist characteristics) and of a guide dog (which we classified as between situative/socio-historical for being a guide (Huang, 2017) and cognitive/constructivist for representing an aid to the blind pupil who she sees as being someone who makes their own decisions).

### Evolution of the Emotional Metaphors

**CHART 2:**
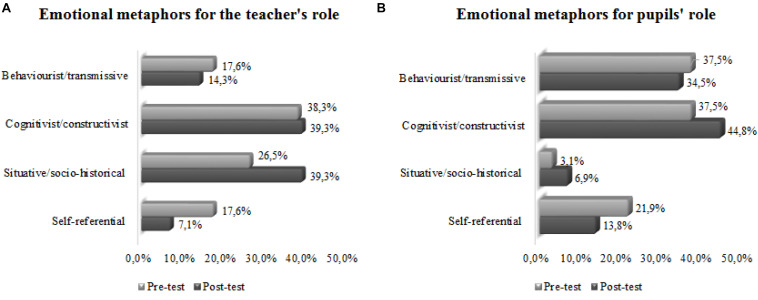
**(A,B)** Comparison of the percentages of emotional metaphors for the teacher’s and pupil’s roles, before and after the practicum.

Regarding the teacher’s role, the participants used 34 emotional metaphors in the pre-test and 31 in the post-test. Chart 2A shows the comparison of the percentages of emotional metaphors in the four categories of study before and after the practicum. In the pre-test, the greatest percentage of teacher emotional metaphors were cognitivist/constructivist, followed by situative/socio-historical, behaviourist/transmissive, and self-referential. In the post-test, there was a large increase in the percentage of situative/socio-historical and a small increase in that of cognitivist/constructivist, and decrease in the behaviourist/transmissive and self-referential.

Regarding the pupils’ role, the participants used 33 emotional metaphors in the pre-test and 29 in the post-test. Chart 2B presents the comparison of the percentages of emotional metaphors in the four categories of study before and after the practicum. In the pre-test, the greatest percentages of pupil emotional metaphors were behaviourist/transmissive and cognitivist/constructivist, followed by self-referential, and situative/socio-historical. In the post-test, there was an increase in the cognitivist/constructivist and situative/socio-historical percentages, and decrease in the behaviourist/transmissive and self-referential metaphors.

**CHART 3:**
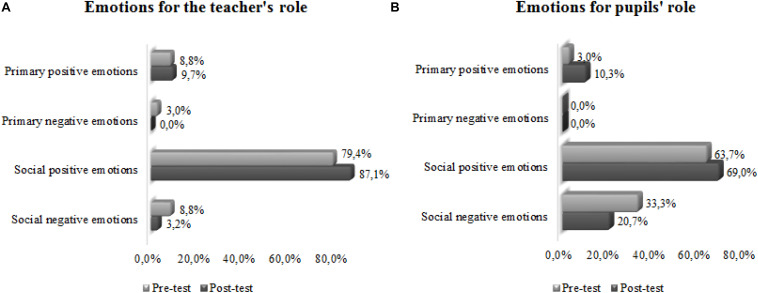
**(A,B)** Comparison of the percentages of emotions for the teacher’s and the pupils’ roles, before and after the practicum.

We would emphasize that, for both the teacher and the pupil metaphors, the greatest number of emotions were the positive social ones, which increased after the practicum (Charts 3A,B). No neutral emotions were identified. We also identified metaphors reflecting primary emotions: positive like happiness (the sun that gives light and heat for the teacher and the flowers in a garden for the pupils) and negative like fear (lamb among wolves for the teacher).

In the following paragraphs, we present some of the participants’ emotional metaphors.

**FIGURE 7:**
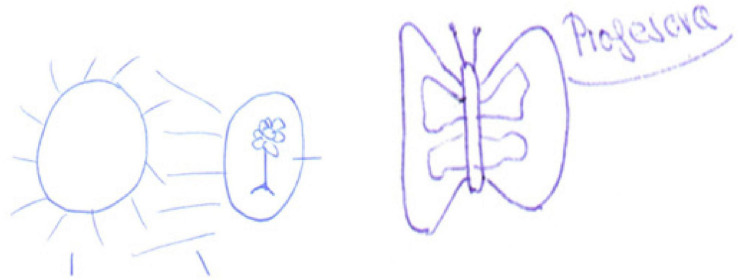
Drawings of the teacher as Sun and butterfly.

Metaphors reflecting positive primary emotions: happiness. Among the teacher, examples are butterfly fluttering among the flowers, a self-referential metaphor drawn by student E24 and the Sun that gives light and heat drawn by student E6 (Figure 7).

Metaphors reflecting negative primary emotions: Fear. For the teacher’s role, we identified the emotion of fear in the metaphor of a lamb among wolves (E3). No negative primary emotions were identified for the pupils’ role.

Metaphors reflecting positive social emotions: Enthusiasm, confidence, protection, etc.

**FIGURE 8:**
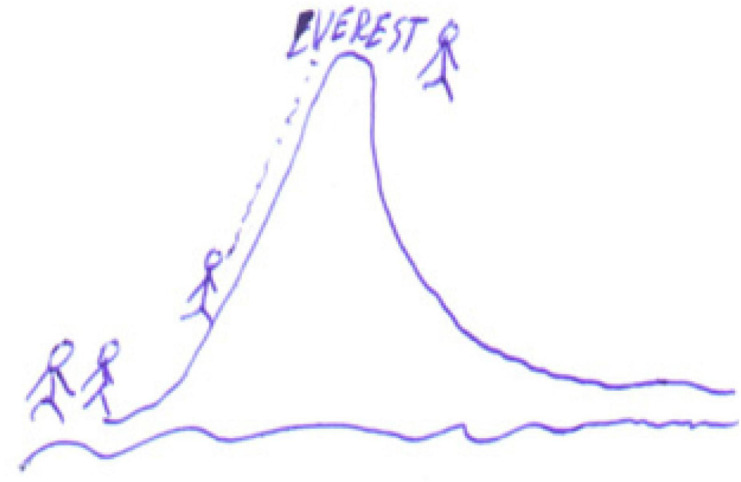
Drawing of the teacher and pupils as mountain climbers.

Enthusiasm, emotion collected by Eren and Tekinarslan (2013). An example is that drawn by student E14 (Figure 8) of the teacher and pupils as mountain climbers who can overcome difficulties and reach the summit. It has a situative/socio-historical meaning, representing teamwork and the teacher as a guide (Cassel and Vincent, 2011; Patchen and Crawford, 2011).

**FIGURE 9:**
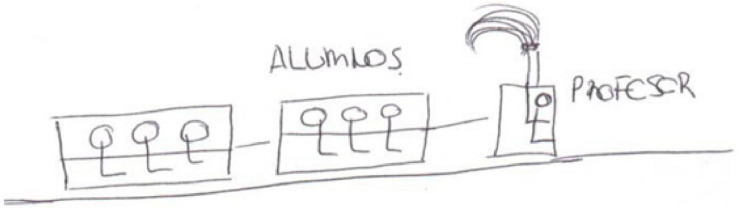
Drawing of the teacher as an engine driver.

Confidence, emotion collected by Plutchik (2001). The drawing of E21 (Figure 9) represents the teacher as an engine driver, who gives the pupils safety and trust.

**FIGURE 10:**
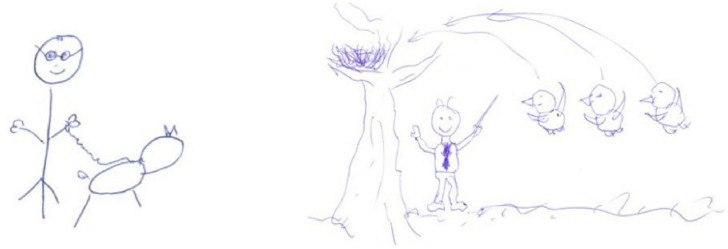
Drawing of the teacher as a guide dog and guiding and protecting fledgling birds.

Protection, emotion related to affection, empathy, etc., emotions collected by Bisquerra (2019). The teacher metaphor of a guide dog can be classified as somewhere between situative/socio-historical (for being a guide) and cognitive/constructivist (for being an aid to the blind person, i.e., the pupil, who nevertheless makes their own decision). The case is similar to the teacher metaphor of someone who takes care of and guides fledgling birds (Figure 10) when they leave (E15) or return (E19) to the nest.

**FIGURE 11:**
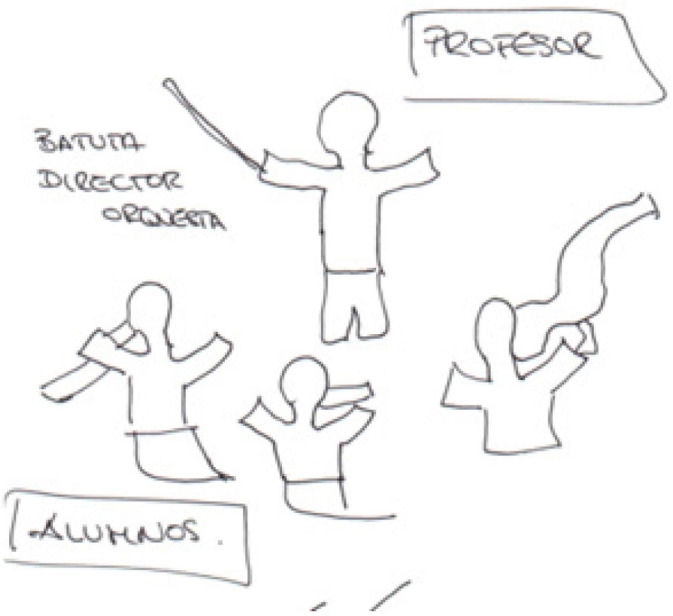
Drawings of the teacher as an orchestra conductor and the pupils as musicians.

Stimulus, motivation. Several participants identified the teacher with being some sort of a guide to motivate their pupils (Thomas and Beauchamp, 2011). Student E8, for instance, sees the teacher as an orchestra conductor (Ben-Peretz et al., 2003; Leavy et al., 2007; Seferoglu et al., 2009); thus, reflecting these stimulus and motivation emotions (Figure 11), with the conductor directing a group of musicians but each pupil playing a different instrument. ‘*The orchestra conductor is the leader who is aware of individual differences and needs, and through successful collaborative work both parties enhance a mutual goal*’ (Akar and Yildirim, 2009, p. 406).

Metaphors reflecting negative social emotions: Intimidation, insecurity, control, etc.

**FIGURE 12:**
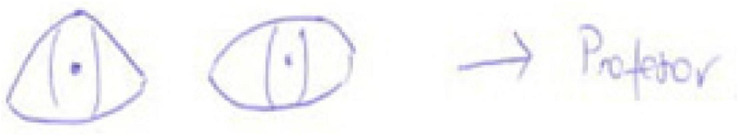
Drawings of the teacher’s killer look.

Intimidation, hostility, emotions collected by Bisquerra (2019). The teacher metaphors expressed by E2 of a killer look (pre-test, Figure 12) and a wolf’s stare (post-test) have a high degree of aggressiveness and are coherent with the pupil metaphor expressed by this participant – as bugs that never stop moving about.

**FIGURE 13:**
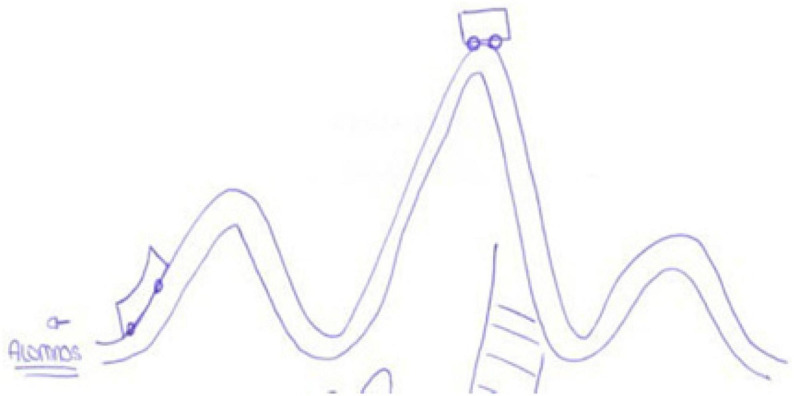
Drawings of the roller coaster as metaphor for the pupils.

Insecurity, emotion collected by Bisquerra (2019). This emotion is associated with E7’s pupil metaphor of the roller coaster (Figure 13) as reflecting the changes in character that adolescents undergo in their everyday lives.

## Discussion and Conclusion

The study shows that all the participants were able to conceptualize both the pupils’ role and their own role as future teachers in the form of metaphors. Their written texts and their pictures were both found to be extraordinary tools to help make the metaphors’ meanings concrete. In their explanations of the reasons for identifying with their personal metaphors, the senses of the writing and the drawing most often matched; however, in those cases in which there were differences, we consider that the drawing better represented the metaphors’ meaning (Weber and Mitchell, 1996; Markic and Eilks, 2015).

In both questionnaires, before and after the practicum, the greatest percentage of teacher role metaphors corresponded to the cognitivist/constructivist category, followed by behaviourist/transmissive in the pre-test and situative/socio-historical in the post-test. In the post-test, we observed increased percentages of cognitivist/constructivist and situative/socio-historical metaphors and a decrease in the behaviourist/transmissive. After a training program, Leavy et al. (2007) indicate an increase in cognitivist/constructivist metaphors and a decrease in behaviourist/transmissive. In addition, Alger (2009), finds that older teachers start teaching with teacher-centred metaphors, and during their careers, some switch to student-centred metaphors. The greatest number of teacher metaphors that belonged to the cognitivist/constructivist category is coherent with the results of another study with prospective secondary school educational guidance teachers (Mellado et al., 2016), but not with the findings of a study of prospective secondary school science teacher (Mellado et al., 2017b) that express a greater number of behaviourist/transmissive metaphors. Among the metaphors most indicated for the teacher role are that of the open book (behaviourist/transmissive), the farmer-sower (cognitivist/constructivist), and the guiding light (situative/socio-historical).

The greatest percentage of pupil role metaphors pre-test corresponded to the behaviourist/transmissive category, followed by cognitivist/constructivist; however, in the post-test, the greatest percentage of pupil metaphors corresponded to cognitivist/constructivist, followed by behaviourist/transmissive and self-referential. In both questionnaires, the lowest percentage of pupil role metaphors corresponded to the situative/socio-historical category, a result that coincides with samples of other specialities (Mellado, 2017). For the pupil metaphors in the post-test, there was an increase in the percentage of cognitivist/constructivist and behaviourist/transmissive and a decrease in the self-referential, with the situative/socio-historical metaphors showing just a slight increase. For the role of the pupils, the metaphor of a sponge (Saban, 2010) was the most common. Animal metaphors were also common for pupils, many of them classified as self-referential: ant, cicada, fly, cow, pig, sheep, snail, turtle, lynx, fox, owl, etc.

There are differences between the metaphors of the role of the teacher and those of the student body, with the teacher generally representing more student-centred models. In some cases, there is coherence between the metaphors of the teacher and those of the student: for example, the guiding lighthouse corresponds to the ships guided by the lighthouse, the gardener corresponds to the seed that germinates and grows, and the water tank corresponds to the garden that receives the water. However, in other cases, there is a lack of coherence, as Strugielska (2008) has already observed: the conductor does not correspond to the hair that grows or the light that illuminates does not correspond to puppets.

A result that coincides with previous studies (Mellado et al., 2012, 2016, 2018) is that the metaphors were not associated with specific content, but were an expression of a general vision of teaching and the role of the teacher, formed from the students’ own experiences as pupils at school (Briscoe, 1991). This is coherent with the finding of Stofflett (1996) that teachers develop metaphors grounded in their personal histories as learners and educators, and that their metaphors are not associated with subject matter.

Comparison of the two sets of results showed that 63.0% of participants for the teacher’s role and 81.5% for the pupils’ role did not change their metaphors and associated models. This result is consistent with the results of previous empirical studies that analyse similar samples of secondary school teachers in training in other specialties, such as science or educational guidance, in which 76.7 and 66.6% did not change the metaphors for the teacher’s role, respectively (Mellado et al., 2016, 2018). These findings are indicative of the difficulty the prospective teachers have in changing their metaphors and associated teaching models because these are already firmly settled when they begin their Teacher Education Master’s course and remained uninfluenced by either the course or the practice teaching. Shaw and Mahlios (2011) reported that most of the subjects in their studies maintained their metaphors unchanged over the years of their teacher education.

However, with respect to those who did change, 29.6% participants showed a progressive change for the teacher’s role and only 7.4% a regressive change. Tannehill and MacPhail (2014) noted that several pre-service teachers, as a result of experience, coursework, and peer discussion, moved from initially viewed themselves as transmitters of knowledge towards a more constructivist notion of teaching and learning. Furthermore, Akar and Yildirim (2009), Löfström and Poom-Valickis (2013), Kavanoz (2016), and Seung et al. (2011) found that when changes occur the metaphors tended to express more pupil-centred educational models. For the pupils’ role, 14.8% participants showed a progressive change and 3.7% a regressive change. These findings show that, although most of the participants did not change their metaphors and associated models, there was still a relevant fraction of them who underwent a progressive evolution towards more pupil-centred educational models. During the Master’s degree, it is the first time that these pre-service teachers have had contact with education or psychology subjects and they have been in teaching practices, but in this research, we do not have data to determine the specific causes that have stimulated or hindered the changes in the metaphors and associated educational models.

In the pre-test, the greatest percentage of teacher emotional metaphors corresponded to the cognitivist/constructivist, followed by situative/socio-historical, and behaviourist/transmissive. The emotional metaphors corresponded to the cognitivist/constructivist, and they are also the majority in secondary pre-service teachers of other specialties (Mellado et al., 2017b). In the post-test, there was a major increase in the percentage of situative/socio-historical metaphors, a small increase in the cognitivist/constructivist, and a decrease in the behaviourist/transmissive. In the pre-test, the greatest percentage of pupil emotional metaphors corresponded to the behaviourist/transmissive and cognitivist/constructivist, followed by self-referential and situative/socio-historical. In the post-test, there was an increase in the percentages of cognitivist/constructivist and situative/socio-historical and a decrease in the percentage of behaviourist/transmissive metaphors.

For both the teacher and the pupil metaphors, the positive social emotions were the most frequent and they increased after the practicum, coincident results for pre-service secondary teachers of other specialties, such as science, technology, and Educational Guidance (Mellado, 2017). The positive primary emotions also increased after the practicum. For the teacher metaphors, only the negative primary emotion of fear was expressed, and this was in the pre-test, which disappeared after the practicum. For the pupil metaphors, there were no negative primary emotions, but more negative social emotions were expressed than for the teacher, although in both cases, these declined after the practicum.

With respect to the study’s implications, it would have been interesting to contrast our interpretation of the metaphors with those of the teachers themselves. It will also have to analyse not only what teachers say but also what they do in their classes to contrast their declared metaphors and models with those that can be observed in their classroom practice. It would also be necessary to analyse the causes of changes or permanence of the metaphors, as well as the cognitive and emotional factors that stimulate or hinder the changes in metaphors. In our study we do not have data on whether the changes in the associated metaphors or models were due to the information and reflection about metaphors made in class with groups of the sample or whether they were due to other factors, such as the theoretical subjects of the Master’s degree, or the practicum.

Future research will have to look more deeply into the possibilities of intervention. As Briscoe (1991) asserted, pre-service science teachers’ reflection on their own metaphors during practices by contrasting them with those of expert tutors, who have innovative models focused on student learning, facilitate prospective teachers to develop their own didactic knowledge of the content. However, there should be more connection and coherence between theory and practice in the Master’s Degree (Allen and Wright, 2014). The practicum may be a great source of professional knowledge, which should stimulate and provide feedback to the metacognitive reflection of the entire Master. However, the finalist teaching practices programmed in the Master of our sample do not meet this objective because they seem to be conceived as a mere application of previous theoretical modules. At the secondary education level, the academist models of teacher education, which are centred on the knowledge of the material to be taught with only a bit of pedagogical knowledge and some teaching practice tacked on at the end are not the most appropriate.

## Data Availability Statement

The raw data supporting the conclusions of this article will be made available by the authors, without undue reservation.

## Ethics Statement

The studies involving human participants were reviewed and approved by the Commission of Bioethics and Biosecurity of the University of Extremadura (Approval No. 158/2020). Written informed consent from the patients/participants was not required to participate in this study in accordance with the national legislation and the institutional requirements.

## Author Contributions

LM conceived and designed the article, collected the data, analyzed and interpreted the data, and wrote the manuscript. LP conceived and designed the article, analyzed and interpreted the data, and wrote the manuscript. SS-H collected the data and analyzed and interpreted the data, and wrote the manuscript. MB collected the data, analyzed and interpreted the data, and wrote the manuscript. All authors contributed to the article and approved the submitted version.

## Conflict of Interest

The authors declare that the research was conducted in the absence of any commercial or financial relationships that could be construed as a potential conflict of interest.
